# Hypoglycemia Among Young Children Presenting With Metabolic Acidosis

**DOI:** 10.7759/cureus.72232

**Published:** 2024-10-23

**Authors:** Ali H Ahmad, Kirstin Henley, Jessica Asencio, Ajay Gupta, Balagangadhar Totapally

**Affiliations:** 1 Pediatric Critical Care, University of Texas MD Anderson Cancer Center, Houston, USA; 2 Pediatric Critical Care, Baylor College of Medicine, San Antonio, USA; 3 Pediatric Critical Care, Los Angeles County Harbor-UCLA Medical Center, Los Angeles, USA; 4 Pediatric Oncology, Roswell Park Comprehensive Cancer Center, University at Buffalo Jacobs School of Medicine and Biomedical Sciences, Buffalo, USA; 5 Pediatric Critical Care, Nicklaus Children's Hospital, Herbert Wertheim College of Medicine, Miami, USA

**Keywords:** gastroenteritis, high anion gap metabolic acidosis, hypoglycemia, normal anion gap metabolic acidosis, vomiting

## Abstract

Objectives

Metabolic acidosis in children can present with varying degrees of severity, duration, and etiology. A minor illness can lead to hypoglycemia and ketosis in young children due to poor metabolic reserve. We aimed to study the etiology and type of metabolic acidosis, associated electrolyte abnormalities, and mortality rate in children presenting with metabolic acidosis.

Methods

We performed a retrospective review of young children aged 1-71 months from January 2014 to March 2015 presenting with metabolic acidosis. The demographic data and laboratory values were extracted. We compared the demographic and clinical characteristics of children presenting with metabolic acidosis with and without hypoglycemia.

Results

The most common diagnoses were acute gastroenteritis (AGE) and vomiting with dehydration. Hypoglycemia was present in 28% of patients, with an overall mortality rate of 2%. Children with acidosis and hypoglycemia tend to be older (42.0 (IQR:20.5-55.5) vs 18.5 (IQR: 7.0-46.3) months; p<0.01), more likely to have diagnoses of vomiting or AGE (67% vs 24%; p<0.01), and have a shorter hospitalization (3 vs 5 days; p<0.05) with no mortality (0% vs 2.6%; p=0.38) compared to children with metabolic acidosis and no hypoglycemia.

Conclusions

Hypoglycemia is common among children presenting with metabolic acidosis. Hypoglycemia should be considered in young children initially presenting with metabolic acidosis. This will help structure our initial management protocols for the pediatric population.

## Introduction

Metabolic acidosis in children can present in varying degrees of severity and duration and with a wide range of etiologies. A child can appear seemingly healthy, as is seen in idiopathic ketotic hypoglycemia, which is the most common form of hypoglycemia in children above 6 months of age, traditionally resolves by 9 years of age, and can also be accompanied by metabolic acidosis [1]. However, other presentations of metabolic acidosis can be severe and contribute to catastrophic hemodynamic collapse secondary to sepsis, toxin ingestion, or inborn errors of metabolism. Metabolic acidosis can be chronic, such as in renal tubular acidosis, or can present as an acute medical problem, such as lactic acidosis secondary to hypoxemia or end-organ hypoperfusion.

Adverse clinical effects of metabolic acidosis on various organ systems have been observed in the pediatric population. Neurological signs of lethargy and confusion can be the primary presenting symptoms of a child with acute metabolic acidosis [2]. Myocardial depression and lethal arrhythmias can occur with severe acute acidosis in children [3]. In the chronic phase, children can face medical complications, including poor growth and development, insufficient bone mineral density, and nephrocalcinosis. As a result, the proper identification and intervention to treat the underlying etiology of metabolic acidosis in the pediatric population becomes paramount and must be managed promptly.

Four mechanisms can help explain the development of metabolic acidosis [4]. The first mechanism involves endogenously increasing acid generation, as seen in lactic acidosis. This can also occur with exogenous intake of acid or acid-producing molecules, such as with propylene glycol toxicity. The body's total acid increases in these mechanisms, causing metabolic acidosis. The second mechanism of metabolic acidosis can be due to a loss of bicarbonate, occurring through the gastrointestinal tract (commonly seen with diarrheal illnesses), or drainage from the small bowel or pancreas [2]. The loss of bicarbonate can also have renal origins, such as those seen in acute kidney injury, and intrinsic renal disease, such as proximal renal tubular acidosis. Proximal renal tubular acidosis is characterized by decreased absorption of bicarbonate in the proximal portion of the renal filtration system, which can contribute to metabolic acidosis. In this category, pediatric patients can also experience a loss of the bicarbonate precursor or inhibition of bicarbonate reabsorption, as seen with diuretics such as carbonic anhydrase inhibitors. The third mechanism responsible for metabolic acidosis involves decreased excretion of acid, such as that seen in distal renal tubular acidosis, also known as Type 1 renal tubular acidosis. This type of acidosis is defined by the inability of the distal nephron to secrete hydrogen ions, therefore causing metabolic acidosis [4]. Finally, bicarbonate dilution can also cause metabolic acidosis, most commonly seen due to high volume infusion of intravenous saline solutions with large chloride anion content, which can lead to metabolic acidosis [5].

Traditionally, metabolic acidosis has been classified as high anion-gap metabolic acidosis (HAGMA) or normal anion-gap metabolic acidosis (NAGMA), depending on whether the anion gap (AG) is increased or not [6,7]. Once a patient is identified as having an increased anion gap, the differential diagnosis will become focused on causes of HAGMA, such as uremia, ketoacidosis, lactic acidosis, and toxicity from ingestion. Children with HAGMA can have additional NAGMA or metabolic alkalosis in mixed gap acidosis. This classification is helpful clinically for etiologic determination.

Hypoglycemia can be a critical associated feature of pediatric metabolic acidosis. It must be promptly detected due to the risk of neurological damage and morbidity that can ensue if left untreated. The level that is considered pathologically hypoglycemic varies by study. Papini et al. found that hypoglycemia, defined by blood glucose between 30 and 45 mg/dL, was commonly associated with gastroenteritis or other infectious etiologies causing prolonged fasting in approximately 86% of their study population and was more prevalent than previously thought [8]. Recommendations from the Pediatric Endocrine Society for Evaluation and Management of Persistent Hypoglycemia in Neonates, Infants, and Children defined clinical hypoglycemia as when plasma glucose is low enough to cause signs and symptoms of impaired brain function, typically between 55-65 mg/dL [9]. Pediatric patients with plasma glucose levels less than 55 mg/dL may present with autonomic symptoms such as diaphoresis and hunger, whereas plasma glucose levels less than 30 mg/dL may cause significant neuroglycopenic symptoms defined by weakness, confusion, coma, and even death [10].

There is limited literature detailing the frequency of different causes and consequences of metabolic acidosis, as well as the occurrence of ketotic hypoglycemia in young children upon hospital admission. This study aimed to assess the demographic profile, underlying causes, and prognosis of children aged 1 month to 71 months presenting with metabolic acidosis at an urban pediatric hospital. Additionally, it aims to further characterize children who present with metabolic acidosis and hypoglycemia.

## Materials and methods

Subjects

We conducted a retrospective chart review of children aged 1-71 months presenting to the emergency room at Nicklaus Children’s Hospital with metabolic acidosis for 15 months from January 2014 to March 2015. For our study, metabolic acidosis was defined as a serum bicarbonate <16 mEq/L [11]. Local IRB approval was obtained, and information technology specialists identified patients who met the above criteria using hospital electronic medical records. The first documented laboratory values, either in the emergency department or upon admission to the hospital, were used for analysis. Only children with serum bicarbonate of <16 mEq/L in their first blood work were included in the study. Children who developed metabolic acidosis later during their hospitalization were excluded from the study. We also reviewed the admission and discharge diagnoses, serum electrolyte panels, serum blood glucose, AG, blood gas values, urine ketones when available, hospital length of stay (LOS), and mortality. Gap acidosis was defined as AG >14 mEq/L [12,13]. AG was calculated using the formula Na-Cl-HCO_3_. Hypoglycemia was defined as blood glucose less than 60 mg/dL [9]. Delta gap was calculated using the formula: Na-Cl-36 for children with increased AG [13]. Based on the AG and delta gap, metabolic acidosis was classified as pure NAGMA, pure HAGMA (with delta gap 0-6 mEg/L), NAGMA and HAGMA (delta gap <0 mEq/L), and HAGMA and metabolic alkalosis (delta gap >6 mEq/L).

Statistical analysis

Patient demographics and clinical characteristics were described and analyzed using means and medians for continuous variables and percentages for categorical variables. Inferential statistics were done using the T-test (or Mann-Whitney U test for non-parametric data) to compare continuous variables and the Chi-square test for categorical variables. We used SPSS Version 28 (IBM Corporation, Armonk, NY) for statistical analysis.

Informed consent and data security

The Institutional Review Board at Nicklaus Children’s Hospital approved the study and waived the requirement for informed consent. Protected health information was initially collected, but names and medical record numbers were replaced with study numbers in the analytical file and were not published or part of the aggregate data. All variables associated with protected health information were de-linked with the final data used for analysis. Information was retained on a password-protected network server. Protocol-specific study numbers were created for each participant, and collected data were maintained in a secure password-protected database on a departmental network server behind a firewall. Complete confidentiality was maintained throughout the study and the preparation and submission of the manuscript.

## Results

A total of 105 children who presented with metabolic acidosis during the 15-month study period were included in the study. The median age of children in the study was 20 months (IQR 10-50). Of the study population, 57.1% (95% CI: 47.1-66.8%) were female. Serum electrolytes and blood sugar were obtained in all children in the cohort. However, all other tests such as blood gases, urine ketones, and blood lactic acid levels, were completed in a select group of the study cohort.

The most common symptoms presented by the children were compatible with gastrointestinal symptoms (41%) consisting of vomiting, which caused secondary dehydration in 15% of cases. Diabetic ketoacidosis occurred in 4.5%. Half of the children presenting with metabolic acidosis, vomiting, and dehydration had hypoglycemia compared to 23.6% of the remaining children in the study group (p<0.05). Children with metabolic acidosis and AGE or vomiting and dehydration are less likely to be admitted (66% vs 91%; p<0.01) and more likely to have hypoglycemia (37% vs 15%; p<0.05) compared to the other children in the cohort. All other diagnoses in this cohort are presented in Table 1.

**Table 1:** The frequency of primary diagnoses in children presenting with metabolic acidosis DKA: Diabetic ketoacidosis

Diagnosis	Number	% (95% CI)
Acute gastroenteritis (Diarrheal illness)	47	44.8 (35.5-54.3)
Vomiting (without diarrhea)	16	15.2 (9.3-23.0)
Respiratory illness (bronchiolitis/pneumonia)	13	12.4 (7.1-19.7)
DKA/metabolic disorders	7	6.7 (3.0-12.6)
Seizures/epilepsy	6	5.7 (2.4-11.4)
Sepsis/infection	5	4.8 (1.8-10.1)
Other illnesses	11	10.5 (5.7-17.4)

The anion gap was increased in 81.9% (95% CI: 73.2-88.7%) of children. The mean bicarbonate level was 13.3 ± 1.92 mmol/L and the mean chloride level was 107.4 ± 6.02 mmol/L. The anion gap and blood urea nitrogen (BUN) were 20.3 ± 5.22 mEq/L and 15.3 ± 8.66 mg/dL, respectively. Blood gas analysis was obtained in 30 patients, and varied amongst arterial blood gas (ABG), capillary blood gas, and venous blood gas. A serum lactic acid level of >2 mmol/L was found in 5 children with metabolic acidosis. Laboratory values are further described in Table 2.

**Table 2:** Laboratory results of children presenting with metabolic acidosis. SD: Standard deviation, BMP: Basic metabolic panel, BUN: Blood urea nitrogen, BG: Blood gas. * Unless otherwise noted

Serum level	Mean ± SD^1^, n = 105*	Median (Interquartile range)
Sodium, mmol/L	141.2 ± 4.58	141 (138 - 144)
Potassium, mmol/L	4.8 ± 0.97	4.7 (4.3 - 5.2)
Chloride, mmol/L	107.4 ± 6.03	107 (104 - 110)
Bicarbonate, mmol/L (from BMP^2^)	13.3 ± 1.92	14 (13 - 15)
Anion gap, mEq/L	20.3 ± 5.2	21 (17 - 24)
Delta gap, mEq/L	-2.1 ± 4.8	-1.0 (-5.0 - +1.0)
Calcium, mg/dL	9.9 ± 1.09	10.1 (9.6 - 10.4)
Glucose, mg/dL	94.9 ± 83.76	75.0 (57.5 - 100.0)
Magnesium, mmol/L, (n = 9)	1.9 ± 0.48	2.0 (1.7 – 2.3)
Phosphorus, mmol/L, (n = 7)	4.9 ± 1.44	5.2 (3.5 – 5.8)
BUN^3^, mg/dL	15.3 ± 8.67	14.0 (10.5 – 19.0)
Creatinine, mg/dL	0.4 ± .13	0.3 (0.3 – 0.4)
BUN/Creatinine ratio	41.5 ± 14.31	40.5 (32.0 – 50.0)
Albumin, mg/dL, (n = 34)	3.9 ± 0.87	4.1 (3.5 – 4.4)
pH, (n = 30)	7.29 ± 0.12	7.30 (7.24 – 7.34)
pCO_2_, mmHg, (n = 30)	32.92 ± 12.46	30.4 (24.0 – 38.5)
Bicarbonate, mmol/L (from BG^4^), (n = 30)	15.71 ± 5.06	16.2 (12.5 – 18.6)
Base deficit, (n = 30)	-9.87 ± 5.79	-10.0 (-13.0 - -4.8)
Lactic acid, mmol/L, (n = 19)	2.3 ± 2.16	1.6 (1.0 – 2.8)

Mixed metabolic acidosis was present in 41.9% of children with metabolic acidosis. The types of metabolic acidosis included pure NAGMA in 18%, pure HAGMA in 40%, NAGMA and HAGMA in 41%, and HAGMA and metabolic alkalosis in 1%. The prevalence of various types of metabolic acidosis was not significantly different with a diagnosis of AGE or the presence of hypoglycemia.

Sixteen (15%) children in our cohort presented with vomiting without diarrhea. Among them, 1 (6.3%) had pure NAGMA, 7 (43.8%) had pure HAGMA, and 8 (50%) mixed HAGMA and NAGMA. None of these children had associated metabolic acidosis. In addition, 8 (50%) had hypoglycemia.

Hypoglycemia was present in 29 patients (28%) of the study cohort. Children with metabolic acidosis and hypoglycemia were found to be older than patients with metabolic acidosis without hypoglycemia (38.4 ± 18.6 vs 25.0 ± 20.2 months; p<0.01). In addition, patients with metabolic acidosis and hypoglycemia were more likely to have diagnoses of vomiting or AGE compared to those patients with metabolic acidosis who were normoglycemic (75.9% vs 48.7%; OR: 3.3, 95% CI: 1.3-8.7; p<0.01). Hypoglycemia was not only frequently found among children with vomiting alone (50%) but was also found in patients diagnosed with acute gastroenteritis (32%). The BUN and BUN-to-creatinine (BUN/Cr) ratios were higher among patients with hypoglycemia (Table 3). Patients with hypoglycemia had a shorter LOS (2 (IQR: 2-2.8) vs 4 (IQR: 2-7) days hospitalized; p<0.05) among hospitalized patients, and no mortality was found in patients with metabolic acidosis and hypoglycemia.

**Table 3:** Children with metabolic acidosis and hypoglycemia vs. children with metabolic acidosis but without hypoglycemia. AGE: Acute gastroenteritis; BUN: Blood urea nitrogen, LOS: Length of stay, IQR: interquartile range,

Characteristic	With hypoglycemia (n=29)	Without hypoglycemia (n=76)	p	T, U, and X^2^ values
Mean age (months)	38.4 ± 18.6	25.0 ± 20.2	<0.01	T = -3.232
Vomiting or AGE(%)	75.9	48.7	OR: 3.3; 95% CI: 1.3-8.7 P=0.012	X^2^ = 6.298 (df 1)
Glucose (mg/dL)	52 (IQR: 46-56)	88.5 (IQR: 72.3-108.8)	<0.001	U = 0
BUN/Creatinine	43.1 ± 11.3	39.2 ± 14.8	0.003	T = -3.044
LOS (days)	2 (IQR: 2-2.8)	4 (IQR: 2-7)	<0.05	U = 684

Urine was tested for the presence of ketones in 41 (40%) children. Out of these 41 children, hypoglycemia and ketonuria were present in 10 (25%). Among the hypoglycemia group, 10 (35%) children were tested for urine ketones, and all were positive for large ketones. Overall, out of 41 tested subjects, ketonuria was present in 31 (76%) children. Ten (24%) children had no ketones. Hypoglycemia was not present in any child who was tested negative for urine ketones.

In our study, there were 105 children presenting with metabolic acidosis, of which 81 (77% (95% CI: 68%-84%)) were admitted to the hospital. Children with AGE were less likely to be admitted ( 65.1% vs 85.5%, OR: 0.32, 95% CI: 0.12-0.82, p=0.015) from the emergency room. A fluid bolus was given to 79 subjects (75% (95% CI: 66%-83%)). Four (3.8%) patients required inotropes, 4 (3.8%) required mechanical ventilation, and 2 required both. The overall mortality rate was 2%. Two children died, one with a diagnosis of intractable epilepsy and the other with a diagnosis of acute respiratory failure.

## Discussion

Metabolic acidosis is a well-known and common acid-base disturbance in critically ill patients [4]. In our study, we describe various types of metabolic acidosis and underlying diagnoses of children under 6 years of age who were seen in an emergency room at a tertiary care children's hospital. Our study demonstrates that a significant portion of children presenting with metabolic acidosis had a diagnosis of AGE (41%) and 28% had initial blood sugar less than 60 mg/dL.

Types of acidosis

Anion gap can be easily calculated using routinely measured serum electrolytes. Although there are several limitations, measuring the anion gap can be useful in identifying various types of metabolic acidosis [7]. Traditionally, metabolic acidosis is classified as NAGMA and HAGMA [7]. In our cohort, the anion gap was high in more than 80% of children with metabolic acidosis. We defined an increased anion gap as >14 mEq/L of uncorrected anion gap. In adult hospitalized patients, 37.6% had an increased anion gap [14]. A total of 16.5% of adults presenting to an emergency room had a high anion gap and those with high anion gap were sicker, needed intensive care admission and had electrolyte abnormalities [15]. The relative proportion of various types of metabolic acidosis depends on the study population, normal values of electrolytes, electrolyte measuring techniques, and correction for unmeasured ions [7]. Despite these limitations, measuring anion gap will be useful in identifying various types of metabolic acidoses and their management. Metabolic acidosis in children with gastrointestinal symptoms is multifactorial in origin. Traditionally, it is taught that AGE presents with NAGMA. However, the metabolic acidosis in AGE can be due to bicarbonate loss from intestinal fluids or due to associated sepsis, acute kidney injury, or starvation ketosis. Hence, our study shows that children with AGE can present with NAGMA or mixed gap acidosis. In children with AGE and acidosis, 74% had normal gap acidosis and 26% had increased anion gap [16]. In another study from a tertiary care hospital in India, 16.5% had mixed acidosis in children with AGE and severe non-gap acidosis [17].

Gastrointestinal symptoms and metabolic acidosis

In our study, gastrointestinal diagnoses or symptoms (AGE or vomiting) were present in more than 50% of children presenting with metabolic acidosis. In a previous study from a children's hospital, the rate of metabolic acidosis in children presenting to the emergency room was found to be highly correlated with the rate of gastrointestinal syndromes [17]. This study defined metabolic acidosis as having bicarbonate levels <20 mEq/L and excluded patients with DKA. In a study from Kenya, 25% of children admitted to hospital were acidotic and children with AGE had the highest prevalence of acidosis [18].

In a prospective study from Norway, metabolic acidosis, defined as bicarbonate values <15 mEq/L, was present in 23%, and hypoglycemia, defined as blood glucose <60 mg/dL, was present in 16% of children presenting with severe AGE [19]. In another study of young children presenting with AGE, metabolic acidosis was present in 25% [20].

Respiratory illness and metabolic acidosis

Acute respiratory illness may present with metabolic acidosis either through the accumulation of lactate or other strong fixed acids or renal loss of bicarbonate as a compensatory mechanism for respiratory alkalosis [21-23]. In our study, approximately 12% of children with metabolic acidosis had primary respiratory illnesses. As we have not measured lactate and ketones in all patients, we cannot report the mechanisms for metabolic acidosis in these patients.

Metabolic acidosis and hypoglycemia

In young children, a third of the patients with metabolic acidosis had hypoglycemia. Studies have reported varying rates of hypoglycemia in young children presenting with AGE, vomiting, or decreased oral intake [19, 24-26]. Hypoglycemia was present in 33.6% of young children presenting with AGE [24]. In contrast, hypoglycemia was uncommon in children presenting with vomiting or decreased oral intake to a Canadian pediatric emergency room [26].

Our study indicated that patients with metabolic acidosis and hypoglycemia were more likely to have diagnoses of vomiting or AGE compared to those patients with metabolic acidosis who were normoglycemic. This is not surprising as previous studies have shown that hypoglycemia is not uncommon in children presenting with AGE symptoms [24]. In addition, up to 10% of children older than one year of age presenting for emergency care with hypoglycemia have a serious underlying condition that would require long-term treatment [27]. Our study found that hypoglycemia was most frequently found among children with vomiting alone versus patients with AGE, similar to previous reports in young children with gastroenteritis, hypoglycemia was associated with more vomiting than diarrhea [24,25]. The importance of this finding cannot be overlooked with the potentially detrimental effects of hypoglycemia in children who present with vomiting and/or AGE symptoms.

Poor energy reserves and exhaustion of glycogen supplies in young children as a means to support the metabolic function of the brain can explain ketogenesis and metabolic acidosis in any situation where poor calorie intake is encountered [28,29]. Children have a higher basal glucose requirement than adults due to a higher metabolic rate and a large brain-to-body mass ratio, thus causing a lower tolerance for fasting in young children. In our study, children who presented with hypoglycemia had higher BUN and BUN/Cr ratios, indicating poor fluid intake. A typical child with diarrhea develops NAGMA due to loss of bicarbonate in the stools. However, poor calorie intake during acute illness, including diarrheal illness, may lead to ketogenesis and additional HAGMA in a young child with low energy reserves. In our cohort, 10% of children with AGE or 50% of children with vomiting and dehydration had hypoglycemia, which supports the mechanism for the genesis of HAGMA in these children. Interestingly, children with hypoglycemia were older in our cohort as reported in another study, and hypoglycemia was more common in over 1 year of age compared to infants [19]. This finding may be related to the feeding patterns of infants compared to older children. Older children may be allowed to go without a feed for extended periods before medical attention is given for acute illnesses.

Idiopathic ketotic hypoglycemia

Idiopathic ketotic hypoglycemia is a known clinical entity seen mostly in young children with poor oral intake, dehydration, and low-calorie reserves. These children present with vomiting due to ketoacidosis and mild hypoglycemia following a minor illness. The pathogenesis of ketotic hypoglycemia is postulated to be due to a lack of glycogen reserves and a deficiency in gluconeogenesis from ketogenic amino acids [30]. Previous studies have suggested a functional defect of hepatic glucose production via gluconeogenesis or decreased gluconeogenic substrates, such as alanine, as mechanisms for the development of ketotic hypoglycemia [31,32]. Recent studies have found that ketotic hypoglycemia is caused by failure to sustain hepatic glucose production and reduced leucine oxidation [33]. Extensive evaluation of the causes of ketotic hypoglycemia was not done in any patients in the present cohort. Although ketotic hypoglycemia was diagnosed in several children in our series, we cannot be certain they all are idiopathic in cause as we have not ruled out other causes.

Children with ketotic hypoglycemia may have undetected ketotic forms of glycogen storage disorders (GSD). Ketotic forms of GSD may not be detected by standard investigations done during an acute episode of hypoglycemia [34]. In our study, none of the children with ketotic hypoglycemia were investigated for ketotic forms of GSD. Several rare metabolic conditions presenting with ketotic hypoglycemia should be considered and investigated based on the clinical presentation [35]. A fructose-1, 6-bisphosphatase deficiency was found to be a cause of recurrent hypoglycemia and metabolic acidosis in one cohort of patients, with patients presenting multiple times in the inpatient setting prior to a diagnosis being made. These rare inborn errors of metabolism require a high degree of suspicion for workup and diagnosis of often treatable conditions [36]. It is also important to separate ketotic from nonketotic hypoglycemia, as nonketotic hypoglycemia can be seen in inborn errors of metabolism such as carnitine transferase deficiency, disorders of free fatty acid metabolism, and hyperinsulinemia syndromes [37]. In addition, adrenal insufficiency can present with non-specific symptoms, including vomiting, difficulty feeding, and hypoglycemia. A high index of suspicion must be maintained to make a diagnosis of adrenal insufficiency in these cases [38].

In young children presenting with metabolic acidosis, anion gap acidosis is typical and is associated with starvation ketoacidosis. Although most children present with reversible causes of metabolic acidosis, potential serious causes of the association between metabolic acidosis and hypoglycemia need to be considered.

There are several limitations to this study. Exhaustive investigations of the causes and mechanisms of metabolic acidosis were not done in all subjects. This was a single-center study, and follow-up information was not available. Additionally, blood gases, urine ketone information, and blood lactate levels were not obtained in all patients. As urine was tested for ketones in less than half of the subjects, ketosis with hypoglycemia may have been much more common in children presenting with metabolic acidosis than we are reporting.

The strength of our study is that we have included all children under six years who presented to an emergency room at an urban children’s hospital during the study period. Our study describes the etiology, types of metabolic acidosis, course, and hospital outcomes of these patients.

## Conclusions

Hypoglycemia is a common association among young children presenting with metabolic acidosis and can frequently be seen in young children presenting with acute gastroenteritis or emesis alone. Anion gap acidosis is the most frequent type of metabolic acidosis, even in children with AGE, and starvation ketosis is the most likely explanation for this. Our study population's mortality rate was low, and the most common causes of metabolic acidosis were reversible. However, the potential for significant pathologic organ system effects of hypoglycemia and metabolic acidosis cannot be understated. Ketotic hypoglycemia should be considered in any young child presenting with gap acidosis, and appropriate treatment and intervention, including rehydration, correction of acid-base imbalance, and treatment of the underlying condition, should be implemented to correct this metabolic acidosis.
